# Impact of natural betaine supplementation on rumen fermentation and productive performance of lactating Damascus goats

**DOI:** 10.1007/s11250-023-03524-4

**Published:** 2023-03-18

**Authors:** Wafaa Mostafa Ali Ghoneem, Reham Roshdi Ali El-Tanany

**Affiliations:** Animal Production Department, Faculty of Agriculture, Cairo University, 12613 Giza, Egypt

**Keywords:** Betaine, Digestibility, Fatty acids profile, Lactating goats, Lactation performance

## Abstract

Two natural betaine sources; dehydrated condensed molasses fermentation solubles (Bet1) and Betafin®, a commercial anhydrous betaine extracted from sugar beet molasses and vinasses (Bet2); were used to investigate their impact on rumen fermentation parameters and lactation performance of lactating goats. Thirty-three lactating Damascus goats, with an average weight of 37 ± 0.7 kg and their age ranged from 22 to 30 months (2^nd^ and 3^rd^ lactation season), were divided into three groups, each group contained 11 animals. The control group (CON) was fed ration without betaine. While the other experimental groups were fed a control ration supplemented either with Bet1 or Bet2 to provide a 4 g betaine/kg diet. Results confirmed that betaine supplementation improved nutrient digestibility and nutritive value, and increased milk production and milk fat contents with both Bet1 and Bet2. Significant increases in concentration of ruminal acetate were observed in betaine-supplemented groups. Goats fed dietary betaine non-significantly recorded higher concentrations of short and medium-chain fatty acids (C4:0 to C12:0), and significant lower concentrations of C14:0 and C16:0 in milk. Also, both Bet1 and Bet2 non-significantly decreased the blood concentrations of cholesterol and triglycerides. Therefore, it could be concluded that betaine can improve the lactation performance of lactating goats and produce healthy milk with beneficial characteristics.

## Introduction

Betaine, also called trimethylglycine or betaine anhydrous, is a natural by-product resulting from sugar beet processing (Fernández et al. 2009a). It acts as an osmolyte when the cell is exposed to osmotic stress and helps in reducing dehydration, stabilization of protein structure, and maintaining enzyme function (Monteiro et al. 2017). Also, it serves as a methyl donor in the animals, which increases methionine concentration (Peterson et al. 2012; Monteiro et al. 2017), and affects many functions in the animal body such as hepatic function, growth, and lactation (Ratriyanto et al. 2009). Additionally, it decreases the concentration of homocysteine, the amino acid that is associated with heart diseases (Peterson et al. 2012). The role of betaine as a methyl donor, confirms its participation in energy, and protein metabolism (Eklund et al. 2005; Poolthajit et al. 2021), and its importance to nervous and cardiovascular systems (Fernández et al. 2009a). Moreover, it helps in polyamide synthesis, which activates the immune defense system (Virtanen and Rumsey 1996; Fernández et al. 2009a).

The ability of betaine to alter rumen fermentation by increasing the acetate concentration was recorded by Wang et al. (2010) and Monteiro et al. (2017). Moreover, higher milk fat content was observed with betaine supplementation to diets of dairy cows (100 g/d) or dairy goats (4 g/kg diet) (Wang et al. 2010; Fernández et al. 2009a), respectively. However, no effect of betaine supplementation was detected on milk fat content by Fernández et al. (2009b), there were significant increases in medium-chain FA concentrations. Furthermore, when betaine was added to the diet, increases in milk yield were demonstrated either in dairy cows (Wang et al. 2010; Peterson et al. 2012; Dunshea et al. 2019; Cheng et al. 2020; Shah et al. 2020; Lewis et al. 2022) as well as in lactating goats (Fernández et al. 2004a, b, 2009a). However, no significant differences in both milk yield and composition with betaine supplementation were recorded by Davidson et al. (2008), Monteiro et al. (2017), Tsiplakou et al. (2017), Wang et al. (2019) and Williams et al. (2021). However, betaine supplementation in rations of lactating Naeini ewes or lactating buffaloes increased milk yield and fat percentage, there were no significant effect on milk protein and lactose contents (Nezamidoust et al. 2012; Shankhpal et al. 2019).

The aims of this study were to investigate the effect of two natural betaine sources on digestibility, nutritive value, lactation performance, ruminal fermentation pattern, and blood metabolites in Damascus lactating goats.

## Materials and methods

The current study was performed in January 2020 at Sheep and Goats Unit, Agriculture Experimental and Research Station, Faculty of Agriculture, Cairo University. Chemical analyses were done at laboratories of the Animal Production Department, Faculty of Agriculture, Cairo University, and National Research Center, Giza, Egypt.

## Experimental animals and rations

Thirty-three lactating Damascus goats, with an average weight of 37 ± 0.7 kg, and their age ranged from 22 to 30 months (2^nd^ and 3^rd^ lactation season), were divided into three balanced groups (11 animals each) after 7 days of parturition according to their weight and milk production (1200 ± 25 g). Goats were housed with their kids and fed in individual pens. The period after morning feeding till evening feeding, all animals for each group were left together in the stall. The experimental groups were randomly given one of the experimental rations. The lactation trial lasted for 60 days. Every 2 weeks, Milk yield was recorded for 3 successive days and milk samples were collected once (Abd El-Hakeem et al. 2021). The lactation trial was followed by 4 days of digestion trial and samples collection of feces, blood, and rumen liquor.

In the current study, two natural betaine sources; Bet1 (dehydrated condensed molasses fermentation solubles (CMS) with 12.7% betaine concentration) and Bet2 (Betafin®, a commercial anhydrous betaine extracted from sugar beet molasses and vinasses with 93% purity) were used. Betafin® (Danisco Animal Nutrition) and dehydrated (CMS) by-products were obtained from MULTIVETA Egypt Trade Company.

All experimental groups were fed rations at 4% of their live weight at the ratio of 40% Egyptian clover (*Trifolium alexandrinum*): 60% concentrate feed mixture (CFM) to cover the total requirements confirmed by NRC (2007). The control group (CON) was fed ration without betaine. While, the other experimental groups were fed a control ration either with Bet1 or Bet2 to provide 4 g betaine/kg diet.

The concentrate feed mixture consisted of 55% yellow corn, 25% wheat bran, 7.5% soybean meal, 10% sunflower meal,1.5% limestone, 0.6% NaCl and 0.4% premix. Each 3 kg of premix contained: 7,000,000 IU (Vit A); 1,500,000 IU (Vit D3); 30,000 mg (Vit E); 60,000 mg (Zn); 60,000 mg (Mn); 50,000 mg (Fe); 20,000 mg (Cu); 1000 mg (I); 250 mg (Co); 300 mg (Se) and up to 3 kg (CaCO3). The chemical composition of CFM, clover (C), and the experimental rations are illustrated in Table 1.

**Table 1:** Chemical composition of the concentrate feed mixture, clover, and experimental ration

Item	Feedstuffs		*Experimental ration
	C	CFM	
DM	90.19	90.83	90.57
Chemical composition, % (DM basis)			
OM	88.16	95.88	92.79
Ash	11.84	4.12	7.21
CP	13.86	13.07	13.39
EE	1.66	2.35	2.07
CF	27.05	5.68	14.23
NFE	45.59	74.78	63.10
Fiber fraction, %			
NDF	44.14	30.21	35.78
ADF	31.94	8.43	17.83

C: clover. CFM: Concentrate feed mixture. * calculated

DM: dry matter, OM: organic matter, CP: crude protein, EE: ether extract, CF: crude fiber, NFE: nitrogen-free extract, NDF: neutral detergent fiber, ADF: acid detergent fiber

The betaine additives (Bet1 and Bet2) were mixed with CFM daily before feeding. Rations (CFM and clover) were fed individually twice daily at 8 am and 3 pm. Feed intake was measured for each animal daily as the difference between the feed offered and the residual (Shah et al. 2020). Determination of dry matter (DM) content in feed was performed once weekly to calculate dry matter intake (DMI). All animal groups were provided with a free choice of fresh water and mineral blocks.

## Milk sampling

The hand milking method was used to collect milk yield from all goats after removing kids away from their mothers for 12 h (from 7 pm to 7 am). The measured milk yield was multiplied by 2 to calculate the daily milk production as described by Smeti et al. (2015). Then 50 ml of milk samples were collected and stored at (-18 °C) for further analysis.

## Digestion trial

After the lactation trial, all goats were taken for three successive days to perform a digestion trial using the bag technique (Uehara et al. 2015; Kotz et al. 2021; Velásquez et al. 2021). Fecal bags were fixed on goats 2 days before starting digestion trial for adaptation. Bags were emptied two times a day before morning and evening feeding (7 am and 2 pm). The total feces content of each bag were mixed well. Feces were sprayed with sulfuric acid (10%) and frozen. Representative feces samples for each animal were pooled and dried at 70° C/24 h, then samples were grinded and kept for chemical analysis (Abd El-Hakeem et al. 2021).

Nutrient digestibility was determined using the acid insoluble ash (AIA) method as recommended by Lee and Hristov (2013) and calculated by the following formula:

$$Digestion\;coefficient=100-\left[100\times\frac{\%\;idicator\;in\;feed}{\%\;ondicator\;in\;feces}\times\frac{\%\;nutrient\;in\;feces}{\%\;nutrient\;in\;feed}\right]$$

## Rumen liquor sampling

On the last day of the experiment, the stomach tube (Shah et al. 2020; Poolthajit et al. 2021; Ghoneem and Mahmoud 2022) was used to collect rumen liquor samples 4 h post-feeding from the six goats in each experimental group, with excluding the first 50 ml of rumen liquor to prevent saliva contamination. Samples were filtered using three layers of cheesecloth, then the pH of ruminal liquor samples was determined immediately by a digital pH meter. Rumen samples were frozen (-18 °C) and stored for analysis of ammonia nitrogen (NH_3_-N), total volatile fatty acids (TVFA’s) after the addition of ortho-phosphoric acid, or individual VFA after the addition of meta-phosphoric acid (Ramos-Morales et al. 2014).

## Blood sampling

At the end of the experiment and before taking rumen liquor samples, blood samples were withdrawn before the morning feeding via jugular vein in tubes containing EDTA (Ethylene Diamine Tetra Acetic acid) anticoagulant. Blood plasma was separated by centrifugation of the blood at 4000 rpm for 20 min., then samples were frozen at -18 °C for further analysis.

## Feeds and feces analysis

Feces and feedstuff samples were chemically analyzed as described by AOAC (2016). The content of NFE (nitrogen-free extract) was calculated as NFE = [100- (ash% + CP% + CF% + EE%)]. Fiber fractions were estimated as recommended by Van Soest et al. (1991).

## Rumen liquor analyses

Rumen pH values were estimated by using a digital pH meter (HI98103, Hanna instruments Inc., Woonsocket, Rhode Island, USA). The ruminal ammonia–nitrogen (NH_3_-N) concentration was determined according to the modified method by Szumacher-Strabel et al. (2002). The steam distillation method was conducted to measure the ruminal concentration of total volatile fatty acids as described by Wang et al. (2016). Individual volatile fatty acids were measured according to Bush et al. (1979).

## Blood plasma analysis

Blood plasma analysis was done using DiaSys spectrophotometer apparatus (340 to 800 nm wavelength). The concentrations of plasma albumin were estimated by Doumas et al. (1971). The concentrations of plasma creatinine and total protein were determined according to Tietz (2006). The concentrations of plasma urea, glucose, aspartate aminotransferase (AST), alanine aminotransferase (ALT), cholesterol, and total triglycerides were measured as recommended by Reed (2013).

## Milk analysis

Total solids (TS), protein, fat, and lactose were determined in milk samples using infrared spectrophotometer apparatus (Foss Matic 120 Milko-Scan, Foss Q3 183 Electric, Hillerød, Denmark) according to the method of AOAC (1995). The difference between TS and fat content was used to calculate the Solids Not Fat content (SNF). The HPLC (High-Pressure Liquid Chromatography) method was used to determine the milk fatty acids profile and concentrations (Pierre-Alain and Moulin 2016).

## Statistical analysis

The general linear model procedure of SAS (2009) was used to analyze the current data. One way ANOVA procedure was done to analyze nutrient digestibility, rumen parameters, milk, feed intake, and blood data via the following model:

$$\mathrm{Yij}=\upmu +\mathrm{Rij}+\mathrm{Eij}$$

where: μ is the overall mean of Yij; Rij is the treatment effect; Eij is the experimental error. Duncan’s New Multiple Range Test was used to calculate the differences among means.

## Results

### Nutrient digestibility and nutritive value

As shown in Table 2, data indicated that the addition of 4 g betaine/kg diet either from dehydrated condensed molasses fermentation solubles (Bet1) or anhydrous betaine (Bet2) significantly (*P* < 0.05) improved nutrient digestibility of dry matter (DM), organic matter (OM), crude protein (CP), crude fiber (CF), ether extract (EE), nitrogen free extract (NFE), neutral detergent fiber (NDF), and acid detergent fiber (ADF). Digestion coefficients of most nutrient and fiber fractions were non-significantly increased with Bet1 compared with Bet2. Regarding the nutritive value, there were significant (*P* < 0.05) increases in TDN (total digestible nutrients) values, while DCP (digestible crude protein) values were not significantly affected by betaine supplementation.

**Table 2:** Nutrients digestibility and nutritive value as affected by betaine addition

Item	Experimental groups			± SE	p-value
	CON	Bet1	Bet2		
Apparent digestibility, %					
DM	72.40^b^	74.39^a^	73.90^a^	0.31	0.001
OM	74.41^b^	77.63^a^	76.80^a^	0.49	< 0.001
CP	74.17^b^	75.98^a^	75.59^a^	0.28	< 0.001
CF	62.10^b^	65.20^a^	66.49^a^	0.67	< 0.001
EE	65.14^c^	65.74^a^	65.46^b^	0.09	< 0.001
NFE	77.54^c^	81.18^a^	79.76^b^	0.53	< 0.001
Fiber fractions:					
NDF	59.38^b^	65.68^a^	64.82^a^	1.00	<0.001
ADF	52.84^b^	57.28^a^	57.09^a^	0.72	< 0.001
Nutritive values, %					
TDN	70.76 ^b^	73.74^a^	72.96^a^	0.48	0.002
DCP	9.93	10.17	10.12	1.51	0.998

Values with a different superscript in the same row are significantly different (*P* < 0.05). TDN: total digestible nutrients. DCP: digestible crude protein. CON: Control group, Bet1, and Bet2: goat groups supplemented with dehydrated condensed molasses fermentation soluble or anhydrous betaine, respectively to provide a 4 g betaine/kg diet

### Rumen liquor parameters

As shown in Table 3, data concerning rumen fermentation parameters revealed no significant differences among the experimental groups in ruminal pH. Meanwhile, significant (*P* < 0.05) decreases in concentrations of rumen NH3-N were found with both Bet1 and Bet2 rations. However, concentrations of TVFA, acetic, butyric, and acetic: propionic (A:P) ratio were significantly (*P* < 0.05) increased in both Bet1 and Bet2 compared with the control. However, propionate concentration was significantly decreased with betaine supplementation.

**Table 3:** Effect of betaine addition on some of the rumen parameters of lactating goats

Item	Experimental groups			± SE	p-value
	CON	Bet1	Bet2		
pH	6.31	6.09	6.07	0.09	0.532
NH_3_-N, mg/100 ml	24.81 ^a^	21.80 ^b^	20.79 ^c^	0.61	< 0.001
TVFA, mmol/L	50.11 ^b^	53.60 ^a^	54.06 ^a^	0.65	0.001
Acetate, mmol/L	25.96 ^b^	28.11 ^a^	29.05 ^a^	0.49	0.003
Propionate, mmol/L	13.49 ^a^	10.14 ^c^	11.72 ^b^	0.52	0.003
A:P ratio	1.93 ^b^	2.79 ^a^	2.49 ^a^	0.14	0.007
Butyrate, mmol/L	8.05 ^b^	10.33 ^a^	10.01 ^a^	0.44	0.042

Values with a different superscript in the same row are significantly different (*P* < 0.05). CON: Control group, Bet1, and Bet2: goat groups supplemented with dehydrated condensed molasses fermentation solubles or anhydrous betaine, respectively to provide a 4 g betaine/kg diet

### Blood parameters

Data in Table 4 showed that betaine supplementation had no significant effect on concentrations of blood total protein (TP), albumin, globulin, creatinine, ALT, AST, cholesterol, and triglycerides. However, the concentrations of blood urea nitrogen (BUN) were significantly (*P* < 0.05) lower with both Bet1 and Bet2 compared with the control. On the other hand, goats fed Bet2 had significantly (*P* < 0.05) the highest concentration of blood glucose (55.43 mg/dl), with no significant difference between those fed Bet1 (54.53 mg/dl) and control (53.74 mg/dl).

**Table 4:** Effect of betaine addition on blood parameters of lactating goats

Item	Experimental groups			± SE	p-value
	CON	Bet1	Bet2		
Total proteins, g/dl	7.01	7.10	7.05	0.03	0.429
Albumin, g/dl	3.90	3.97	3.89	0.02	0.344
Globulin, g/dl	3.11	3.13	3.16	0.02	0.743
Urea-N, mg/dl	25.65 ^a^	24.27 ^b^	23.98 ^b^	0.27	0.001
Creatinine, mg/dl	0.84	0.83	0.87	0.02	0.658
AST, IU/L	81.60	81.77	80.95	0.66	0.896
ALT, IU/L	11.91	11.68	11.85	0.20	0.912
Glucose, mg/dl	53.74 ^b^	54.53 ^b^	55.43 ^a^	0.28	0.009
Total cholesterol, mg/dl	95.35	94.90	94.92	0.21	0.687
Triglycerides, mg/dl	15.39	14.55	14.90	0.34	0.665

Values with a different superscript in the same row are significantly different (*P* < 0.05). CON: Control group, Bet1, and Bet2: goat groups supplemented with dehydrated condensed molasses fermentation solubles or anhydrous betaine, respectively to provide a 4 g betaine/kg diet. ALT: Alanine aminotransferase and AST: Aspartate aminotransferase

### Milk yield, composition, and feed efficiency

The effects of betaine supplementation on milk yield and composition, dry matter intake (DMI), and feed efficiency are presented in Table 5. Results indicated significant higher (*P* < 0.05) milk and 4% FCM yields for goats fed either Bet1 or Bet2 than those fed control ration, with no significant difference between Bet1 and Bet2. Regarding milk composition, total solids and milk fat % were significantly (*P* < 0.05) increased with dietary betaine, with no significant differences between Bet1 and Bet2. On the other hand, there were no significant differences in the contents of milk protein, lactose, ash, and SNF among the experimental groups. Higher (*P* < 0.05) DMI was recorded with Bet1 (1.31 kg/d) and Bet2 (1.30 kg/d) compared to 1.28 kg/d in control. While betaine supplementation had no significant effect on feed efficiency.

**Table 5:** Effect of betaine addition on milk yield and composition, dry matter intake, and feed efficiency

**Item**	Experimental groups			± SE	p-value
	CON	Bet1	Bet2		
Milk Yield (kg/h/d)	1.24 ^b^	1.36 ^a^	1.33 ^a^	0.02	0.038
4% FCM (kg/h/d)	1.29 ^b^	1.46 ^a^	1.44 ^a^	0.03	0.002
Milk composition, %					
Fat	4.26 ^b^	4.47 ^a^	4.55 ^a^	0.04	0.001
Protein	3.46	3.75	3.65	0.20	0.562
Lactose	4.70	4.71	4.69	0.03	0.986
Ash	0.93	0.91	0.92	0.03	0.974
SNF	9.09	9.36	9.26	0.08	0.433
TS	13.35 ^b^	13.83 ^a^	13.81	0.10	0.047
DMI (kg)	1.28 ^b^	1.31 ^a^	1.30 ^a^	0.01	0.001
Feed Efficiency					
Milk yield/DMI, kg/kg	0.95	1.04	1.02	0.02	0.180

Values with a different superscript in the same row are significantly different (*P* < 0.05)

CON: Control group, Bet1, and Bet2: goat groups supplemented with dehydrated condensed molasses fermentation soluble or anhydrous betaine, respectively to provide a 4 g betaine/kg diet

### Milk fatty acids profile

Data concerning the influence of betaine supplementation on milk F.A. profile (Table 6), indicated no significant effect with betaine supplementation on most F.A. proportions, except for C10:0, C14:0, and C16:0, which showed significant (*P* < 0.05) increases in C10:0 as well as significant decreases in C14:0 and C16:0 with dietary betaine groups (Bet1 and Bet2). However, the total saturated (TSFA), de novo, total unsaturated (TUFA), and total polyunsaturated (TPUFA) fatty acids were not affected by betaine supplementation.

**Table 6:** Milk fatty acids profile as affected by betaine addition

**Item**	Experimental groups			± SE	p-value
	C	Bet1	Bet2		
C4:0	2.35	2.39	2.42	0.06	0.926
C6:0	2.29	2.44	2.38	0.06	0.692
C8:0	2.41	2.90	2.97	0.29	0.487
C10:0	8.29^b^	10.73 ^a^	10.56 ^a^	0.41	0.001
C10:1	0.23	0.28	0.27	0.03	0.119
C11:0	0.15	0.19	0.20	0.03	0.280
C12:0	4.42	5.37	5.31	0.48	0.646
C14:0	9.06 ^a^	8.61 ^b^	8.70 ^b^	0.08	0.035
C15:0	0.72	0.75	0.73	0.04	0.969
C16:0	28.71 ^a^	26.03 ^b^	25.84 ^b^	0.47	0.000
C16:1	1.64	1.57	1.58	0.08	0.939
C17:0	0.75	0.76	1.76	0.57	0.999
C17:1	0.49	0.33	0.31	0.19	0.200
C18:0	7.39	7.69	7.74	0.40	0.944
C18:1	18.12	19.32	19.45	0.57	0.638
C18:2	1.95	1.81	1.83	0.08	0.794
C18:3	0.46	0.41	0.40	0.06	0.908
C20:0	0.14	0.16	0.15	0.01	0.224
TSFA	66.68	68.02	67.76	0.80	0.814
TUFA	22.89	23.73	23.84	0.49	0.115
TPUFA	2.41	2.22	2.23	0.12	0.788
Total de novo	55.12	55.57	55.21	0.55	0.954

Values with a different superscript in the same row are significantly different (*P* < 0.05)

CON: Control group, Bet1, and Bet2: goat groups supplemented with dehydrated condensed molasses fermentation soluble or anhydrous betaine, respectively to provide a 4 g betaine/kg diet. TSFA: total saturated fatty acids, TUFA: total unsaturated fatty acids, TPUFA: total polyunsaturated fatty acids. Total de novo: total of (C4:0, C6:0, C10:0, C12:0, C14:0 and C16:0)

## Discussion

In the present study, higher nutrient digestibility and nutritive value were observed with betaine supplementation either from dehydrated condensed molasses fermentation solubles or anhydrous betaine. In the same line with the current data, the digestibility of DM, OM, CP, and fiber fraction were increased when betaine was added to Holstein dairy cowsʼ rations at either 50, 100, or 150 g/d (Wang et al. 2010), 4 g/kg DM (Cheng et al. 2020) or 15 g/d (Shah et al. 2020). Similarly, digestibility of NDF and ADF were higher when Angus bullsʼ rations were supplemented with 0.6 g betaine/kg DM (Wang et al. 2020). On the other hand, no significant differences in digestibility of DM, OM, EE, NDF, and ADF were detected when a mixture of betaine, biotin, and chromium was added to the rations of feedlot bulls (Poolthajit et al. 2021). The improvement in nutrient digestibility and nutritive value in the current study with betaine supplementation could be explained by the improvement in microbial fermentation of the diet (Eklund et al. 2005), and also may be due to the role of betaine as a methyl donor which maintains the rumen pH (Shah et al. 2020). In the same context, Cheng et al. (2020), Wang et al. (2020) and Liu et al. (2021) recorded increases in the total count of ruminal bacteria and protozoa, and in the activity of ruminal enzymes such as carboxymethyl cellulase, xylanase, cellobiase and protease which may explain the improvement in the digestion with betaine supplementation. A possible positive effect of betaine on energy utilization as a result of its osmolyte properties was recorded by Eklund et al. (2005).

The current results illustrate the ability of betaine to alter the rumen fermentation pattern, as it can be used as a source of methyl groups or available nitrogen in the rumen (Löest et al. 2002). The increases in TVFA concentration and A:P ratio with betaine supplementation could be attributed to the increase in acetate and butyrate and the decrease in propionate concentrations. Consistent with the previous findings, betaine was demonstrated to be metabolized by rumen microbes and converted into acetic acid (Peterson et al. 2012; Cheng et al. 2020), which plays an important role in fat synthesis (Shah et al. 2020). In addition, the increase in concentrations of TVFA and acetate with betaine supplementation may be due to higher NDF and ADF digestibility in the present study, which indicates a higher rate of microbial fermentation (Wang et al. 2010). Rumen microbes can be promoted by betaine supplementation due to their osmoprotective effect (Wdowiak-Wróbel et al. 2013). The decrease in the concentration of ruminal NH3-N and the increase in CP digestibility with betaine supplementation may confirm the improvement in N utilization in the rumen (Wang et al. 2010), which may be attributed to the higher growth of rumen microbes (Cheng et al. 2020; Wang et al. 2020). In agreement with the current results, decreases in ruminal NH3-N, and propionate, and increases in TVFA, acetate, butyrate, and A:P ratio were observed when betaine was added to rations of dairy cows (Wang et al. 2010; Nakai et al. 2013; Shah et al. 2020). In parallel to the present study, Cheng et al. (2020) reported no significant effect on rumen pH, significant increases in TVFA and acetate, and significant decreases in NH3-N when 4 g betaine/kg DM was added to the diet of dairy cows. However, there were no significant effects on ruminal NH3-N, acetic, propionic, butyric acids, and A:P ratio, with significant decreases in TVFA when the ration of feedlot bulls (60 concentrates: 40 roughages) was supplemented with a mixture of betaine, biotin, and chromium (Poolthajit et al. 2021).

In concordance with the present results, no changes were detected in concentrations of blood TP and albumin (Fernández et al. 2009a; Cheng et al. 2020), ALT (Zhang et al. 2014), creatinine (Fernández et al. 2009a) and glucose (Fernández et al. 2009a; Wang et al. 2010; Zhang et al. 2014; Monteiro et al. 2017; Cheng et al. 2020; Poolthajit et al. 2021) with betaine supply. While, Shah et al. (2020) recorded increases in concentrations of blood glucose with betaine supplementation especially when it was added at 15 g/cow/d, and explained that it resulted from the improvement in digestibility and feed intake with betaine supplementation. In agreement with the current data, Zhang et al. (2014) demonstrated decreases in the concentration of BUN when betaine was added at 10, 15, and 20 g/cow/d. That result confirms higher N utilization and retention as a result of betaine supplementation (Zhang et al. 2014). Although concentrations of blood cholesterol and triglycerides were not significantly altered in the current study, they were numerically decreased with betaine supplementation. The decline in blood concentrations of triglycerides and cholesterol may be explained by the increase in fat mobilization (Zhang et al. 2014), which could be promoted by betaine due to its role as a methyl donor (Davidson et al. 2008). In accordance with the current findings, Davidson et al. (2008) showed that the addition of 40 g rumen-protected betaine/d in rations of dairy cows had no significant influence on blood triglycerides and total cholesterol. However, a decrease in the concentration of triglycerides with betaine supply was recorded by Fernández et al. (2009a) when goatsʼ ration was supplemented with 4 g betaine/kg DM in the summer season. This inconsistent effect of betaine on blood concentrations of triglycerides and cholesterol may be attributed to that animals in the current study were not exposed to extraordinary environmental conditions such as heat stress, so the dietary treatment had a limited effect on lipid metabolism (Davidson et al. 2008). The current blood plasma metabolites indicate no adverse effect on goats' health, as the values of blood parameters were within the normal ranges (Mohammed et al. 2016).

Milk yield in the present study was increased by 10 and 7% with Bet1 and Bet2, respectively. In the same trend, the addition of 4 g betaine/kg DM either in goatsʼ ration (Fernández et al. 2004a, b, 2009a) or in dairy cowsʼ ration (Cheng et al. 2020) increased milk yield. Similarly, several studies conducted on dairy cows confirmed higher milk yield with betaine supplementation (Wang et al. 2010; Peterson et al. 2012; Zhang et al. 2014; Monteiro et al. 2017; Dunshea et al. 2019; Shah et al. 2020). Also, an improvement in milk production with 35 and 70 g betaine/cow/d was observed under thermoneutral conditions (Hall et al. 2016). Whereas, no significant effect on milk yield was observed when the ration of multiparous cows was supplemented with 20 g rumen-protected betaine (Davidson et al. 2008). The increase in milk production with betaine supplementation may be a result of the improvement in nutrient digestibility (Wang et al. 2010). Betaine could promote mammary growth which reflects in higher milk production as a result of maintaining cellular osmolarity and promoting cell proliferation (Monteiro et al. 2017). The positive effect of betaine supplementation on mammary gland has been confirmed by several studies, which may be ascribed to the decrease in the milk somatic cells֬ number (Wang et al. 2019, 2020), the reduction in the oxidative damage of the mammary epithelial cells under heat stress conditions (Li et al. 2019) and alleviation of the inflammation cytokines production, which suggests the ability of betaine to be an effective feed additive for mastitis (Zhao et al. 2022). Another possible reason for the positive effect of betaine on nutrient digestibility and animal performance could be due to its effect on improving mitochondrial function via increasing mitochondrial fission and fusion factors, which were demonstrated to have a role in maintaining cell homeostasis and promoting cell survival (Kim 2018). Furthermore, it was confirmed that betaine can stabilize cellular and subcellular membrane through restoring both enzymatic and non-enzymatic antioxidants, and positively regulate the mitochondrial function (Lee 2015; Wen et al. 2021). Therefore, we think that betaine may improve the function of rumen microbes, because part of betaine can be digested and utilized by rumen microorganisms (Nakai et al. 2013).

Regarding milk composition, the increase in milk fat content with betaine supply was reported by several studies. It was indicated that dietary supplementation with 4 g betaine/kg DM significantly increased milk fat % either in lactating Murciano-Granadina goats (Fernández et al. 2004b, 2009a) or in dairy Holstein cows (Cheng et al. 2020). Also, increases in milk fat content with betaine supplementation in Holstein dairy cowsʼ rations were observed by Wang et al. (2010), and Monteiro et al. (2017). However, the findings of Fernández et al. (2004a, 2009b), Peterson et al. (2012), Zhang et al. (2014) and Dunshea et al. (2019) indicated that there were no significant differences in milk fat content with betaine supplementation either in rations of lactating Murciano-Granadina goats, Holstein or Friesian × Holstein cows, respectively. In the present study, the increases in milk fat % with Bet1 and Bet2 (4.47 and 4.55 respectively vs 4.26%), are consistent with the increases in concentrations of ruminal acetate (28.11 and 29.05 mmol/l, respectively) compared with control (25.96 mmol/l). It was suggested that betaine can alter rumen fermentation patterns towards higher acetate concentration that may in turn promote fat synthesis by the mammary gland (Monteiro et al. 2017). The lack of significant effect of betaine supplementation on milk protein and lactose concentrations in the present study agrees with the results recorded by Fernández et al. (2004b, 2009a, b) and Wang et al. (2010). Monteiro et al. (2017) observed a decrease in milk lactose content despite similar concentrations of blood glucose when betaine was added to ration of Holstein dairy cows. They explained the lack of conversion blood glucose to milk lactose by reducing the synthesis rate of lactose by mammary gland as a result of betaine supplementation. Wang et al. (2010) explained the inconsistent effect of betaine supplementation on milk yield or composition as a result of the differences in betaine form and dose or the basal diet content of CP. Increases in DMI were recorded with betaine groups. Similar results were obtained by Zhang et al. (2014), Dunshea et al. (2019) and Shah et al. (2020), which reported significant increases in feed intake with betaine supplementation either in Holstein or Friesian × Holstein dairy cowsʼ ration. However, DMI was not affected by betaine added to rations of Holstein dairy cows in other studies (Wang et al. 2010; Peterson et al. 2012; Monteiro et al. 2017; Cheng et al. 2020). Although there were no significant differences in feed efficiency (FE) between the experimental groups, betaine-supplemented goats showed higher FE being 1.04 and 1.02 kg/kg than control goats (0.95 kg/kg). This non-significant improvement in FE in the current study with betaine supplementation may account for the increase in milk yield. However, higher FE was expected with the high betaine dose used in the present study (4 g/kg diet) in comparison to the other studies. Wang et al. (2010) attributed that result to the variation in form and dose of betaine used or in the basal diet composition. There are several conflicting studies, Davidson et al. (2008), Wang et al. (2010), and Peterson et al. (2012) showed no significant change in FE when dairy cow's ration was supplemented with betaine, while Monteiro et al. (2017) observed a significant increase in FE with betaine supplementation.

Concerning the influence of betaine supplementation on milk F.A. profile, the current data indicated that percentages of individual F.A. were in agreement with the ranges conducted by Alonso et al. (1999). While, the sum of the most important F.A. (C10:0, C14:0, C16:0, C18:0, and C18:1) in goatsʼ milk accounted for 77 or 78% of the total F.A. (Fernández et al. 2004a, b), the current study reported a lower value of 72%. Significantly higher values of capric acid C10:0 (10.73 and 10.56 vs 8.29%) and lower values of myristic acid C14:0 (8.61 and 8.70 vs 9.06%) and palmitic acid C16:0 (26.03 and 25.84 vs 28.71%) were observed in the current study with dietary betaine groups (Bet1 and Bet2), respectively. In the same trend, Fernández et al. (2004a, b) demonstrated significant increases in C10:0 and non-significant decreases in C14:0 and C16:0 when goats were fed a ration containing 4 g betaine/kg DM. Whereas, concentrations of C10:0, C14:0, and C16:0 obtained by Peterson et al. (2012) did not differ when betaine was added to dairy cowsʼ ration. Fernández et al. (2009b) reported health benefits for humans with betaine supplementation as a result of decreasing concentrations of C14:0 and C16:0 due to their hypercholesterolemic effect (McGuire et al. 1997). Although the present data showed no significant differences in short and medium-chain F.A. (C4:0, C6:0, C8:0, C10:1, C11:0, and C12:0), there was an improvement in their concentrations with betaine supplementation. Also, higher but non-significant values of C18:0 and C18:1 and lower values of C18:2 and C18:3 were observed with betaine-supplemented groups. The increase in short and medium-chain F.A. with betaine supplementation is in parallel with the results obtained by Fernández et al. (2004a, b, 2009b). Fernández et al. (2004b) suggested that the increase in short and medium-chain F.A. with betaine supply may be due to the increase in nutrient availability that is needed for F.A. synthesis by the mammary gland. However, the decrease in C18 fatty acids, which are of dietary origin, may be attributed to the higher acetate proportion that affects the concentrations of blood triglycerides and free fatty acids. Besides, the increases in short and medium-chain F.A. with betaine supplementation were found to be related to the hypocholesterolemic effect, which has beneficial properties for the consumersʼ health (Fernández et al. 2009b). In the same context, the total saturated (TSFA), de novo, unsaturated (TUFA), and polyunsaturated (TPUFA) fatty acids were not affected by betaine supplementation. Similarly, no effect on TSFA, and TPUFA was demonstrated by Fernández et al. (2004a, 2009b) for goats fed rations supplemented with betaine. Also, Peterson et al. (2012) reported no effect on TSFA, TUFA, and de novo fatty acids with lower TPUFA concentrations when betaine was added at 25, 50, and 100 g/cow/d.

## Conclusion

Under thermoneutral conditions, betaine supplementation either in form of dehydrated condensed molasses fermentation solubles or anhydrous betaine extracted from sugar beet molasses and vinasses at a 4 g/kg diet, improved both nutrient digestibility and nutritive value, recorded higher milk production for Damascus lactating goats with healthy and beneficial characteristics.

## Data Availability

The raw data for this research will be available from the corresponding author upon request.
